# Telemedicine Use and Health-Related Concerns of Patients With Chronic Conditions During COVID-19: Survey of Members of Online Health Communities

**DOI:** 10.2196/23795

**Published:** 2021-02-18

**Authors:** Lindsey Nicole Horrell, Sara Hayes, Leslie Beth Herbert, Katie MacTurk, Lauren Lawhon, Carmina G Valle, Amrita Bhowmick

**Affiliations:** 1 William F Connell School of Nursing Boston College Chestnut Hill, MA United States; 2 Health Union, LLC Philadelphia, PA United States; 3 Department of Nutrition Gillings School of Global Public Health University of North Carolina at Chapel Hill Chapel Hill, NC United States; 4 Lineberger Comprehensive Cancer Center University of North Carolina at Chapel Hill Chapel Hill, NC United States; 5 Department of Health Behavior Gillings School of Global Public Health University of North Carolina at Chapel Hill Chapel Hill, NC United States

**Keywords:** telehealth, telemedicine, coronavirus, COVID-19, chronic disease

## Abstract

**Background:**

It has been widely communicated that individuals with underlying health conditions are at higher risk of severe disease due to COVID-19 than healthy peers. As social distancing measures continue during the COVID-19 pandemic, experts encourage individuals with underlying conditions to engage in telehealth appointments to maintain continuity of care while minimizing risk exposure. To date, however, little information has been provided regarding telehealth uptake among this high-risk population.

**Objective:**

The aim of this study is to describe the telehealth use, resource needs, and information sources of individuals with chronic conditions during the COVID-19 pandemic. Secondary objectives include exploring differences in telehealth use by sociodemographic characteristics.

**Methods:**

Data for this study were collected through an electronic survey distributed between May 12-14, 2020, to members of 26 online health communities for individuals with chronic disease. Descriptive statistics were run to explore telehealth use, support needs, and information sources, and *z* tests were run to assess differences in sociodemographic factors and information and support needs among those who did and did not use telehealth services.

**Results:**

Among the 2210 respondents, 1073 (49%) reported engaging in telehealth in the past 4 months. Higher proportions of women engaged in telehealth than men (890/1781, 50% vs 181/424, 43%; *P*=.007), and a higher proportion of those earning household incomes of more than US $100,000 engaged in telehealth than those earning less than US $30,000 (195/370, 53% vs 241/530 45%; *P*=.003). Although 59% (133/244) of those younger than 40 years and 54% (263/486) of those aged 40-55 years used telehealth, aging populations were less likely to do so, with only 45% (677/1500) of individuals 56 years or older reporting telehealth use (*P*<.001 and *P*=.001, respectively). Patients with cystic fibrosis, lupus, and ankylosing spondylitis recorded the highest proportions of individuals using telehealth when compared to those with other diagnoses. Of the 2210 participants, 1333 (60%) participants either looked up information about the virus online or planned to in the future, and when asked what information or support would be most helpful right now, over half (1151/2210, 52%) responded “understanding how COVID-19 affects people with my health condition.”

**Conclusions:**

Nearly half of the study sample reported participating in telehealth in the past 4 months. Future efforts to engage individuals with underlying medical conditions in telehealth should focus on outreach to men, members of lower-income households, and aging populations. These results may help inform and refine future health communications to further engage this at-risk population in telehealth as the pandemic continues.

## Introduction

According to the Centers for Disease Control and Prevention (CDC), people with underlying medical conditions (eg, autoimmune disease, chronic respiratory disease, and cancer) are at greater risk of severe illness from COVID-19 than those without pre-existing conditions [1]. Experts have released multiple guidelines to protect the health and safety of at-risk communities during this pandemic, including instructions to socially distance, stay at home as much as possible, and avoid crowds [1-3]. For communities who must continue health care appointments and treatment regimens during the pandemic, telehealth, or electronic communication and information technologies to support health care [4], has been recommended as a feasible intervention to maintain continuity of care while adhering to social distancing and stay-at-home mandates [1,5-9]. Although telehealth use rapidly increased during the initial spread of COVID-19 [10,11], little information is available regarding telehealth uptake among high-risk populations with underlying medical conditions during this pandemic.

Thus, the purpose of this study is to describe the telehealth use, resource needs, and information sources of individuals with chronic conditions during the COVID-19 pandemic. Data for this study were collected from members of online health communities hosted by the digital publishing platform, Health Union, for patients with chronic conditions (eg, communities for those with migraines, lung cancer, chronic obstructive pulmonary disease, or rheumatoid arthritis) [12]. This paper also explores differences in telehealth use among sociodemographic characteristics to identify those who may be in most critical need of support. Given the importance of understanding an audience’s response in health communication evaluation [13] and the fact that increased telehealth efforts are likely to continue both during and beyond the COVID-19 pandemic [11], the information included in this paper may play a helpful role in informing future efforts to engage patients in telehealth practices.

## Methods

### Data Collection

Data for this study were collected in an online survey distributed through Qualtrics Survey Software (Qualtrics International Inc) to members of 26 online health communities hosted by Health Union. Each community provides patients and caregivers a digital platform to learn about their diagnosis and both provide and receive social support from peers and health care providers [12]. Between May 12-14, 2020, the survey was emailed to 98,983 community members who had previously opted into receiving emails from Health Union. The study team also posted information about the survey on the online health community websites owned and operated by Health Union. To complete the survey, respondents had to be 18 years or older, living within the United States, aware of COVID-19, and diagnosed with one of 26 chronic conditions. The survey included 35 questions assessing telehealth engagement, health information sources and needs during this pandemic, concerns about COVID-19, self-reported chronic condition diagnoses, and demographic characteristics. In this survey, telehealth was defined as “virtual appointments with doctors that may take place over video chat.” Upon completion, each participant was entered into a chance drawing to win one of one hundred US $25 e-gift cards.

### Measures

#### Telehealth Engagement and Satisfaction

In this study, telehealth engagement was measured by asking participants to respond “yes” or “no” to the question “Have you had a ‘virtual visit’ (telehealth) with your doctor / healthcare professional in the past 4 months?” To further assess telehealth practices, participants were asked, “You may have heard about virtual appointments with doctors (sometimes called ‘telehealth’) that may take place over video chat. Which statements below about telehealth apply to you?” Participants then indicated all statements that applied to them from a list of eight items such as “My doctor has reached out to me about telehealth,” “I am interested in telehealth but don’t know how to use it,” and “I started using telehealth (or will start) because of the pandemic.” The full list of item responses can be found in Multimedia Appendix 1.

Among those who had participated in a telehealth visit in the past 4 months, satisfaction was assessed by asking participants to rate their agreement with the following three statements: “I had a positive experience using telehealth,” “The technology was difficult to use,” and “I feel like the virtual visit was just as good (or better) than an in-person visit.” Responses were on a 7-point Likert scale (1=completely disagree to 7=completely agree); in these analyses, “agreement” with each statement was defined as selecting a 6 or 7, while “disagreement” was defined as selecting a 1 or 2.

#### COVID-19 Information Sources and Needs

##### COVID-19 Communications With a Health Care Team

To assess how individuals with underlying medical conditions have been gathering information about COVID-19, participants were first asked questions about pandemic-related interactions with their health care team. For example, participants were asked, “In which of the following ways have you communicated with your doctor/healthcare team about the novel coronavirus (COVID-19)?” and prompted to choose from a list of eight item responses (eg, I reached out, my doctor personally contacted me, or we discussed it at a regular appointment). Among those who had communicated with their health care team, participants were asked, “How have you communicated with your doctor/healthcare professional?” (eg, by phone, portal, or email) and “What did you discuss with your doctor/healthcare provider?” (eg, deciding to temporarily stop or skip medications, monitoring for COVID-19 symptoms, or regular discussion or check-in about my current condition and symptoms). Participants were also asked “What, if anything, have you done – or are you planning to do in the near future -- in response to hearing about the novel coronavirus (COVID-19)?” Item responses included “cancel or postpone regular doctor/healthcare visits”; “have a ‘virtual’ visit with a doctor/healthcare professional – ‘telehealth’ – via video chat”; and other items related to health decisions, needs, and information seeking behavior. A complete list of item responses for each of these questions can be found in Multimedia Appendix 1.

##### Patient Concerns During the COVID-19 Pandemic

To assess patient concerns during the COVID-19 pandemic, participants were asked to indicate their agreement with a series of nine statements on a 7-point Likert scale (1=completely disagree to 7=completely agree). The full list of statements can be found in Multimedia Appendix 1, but examples included “Having a chronic health condition makes me feel particularly concerned about the coronavirus,” “I feel like people are not taking the coronavirus seriously enough,” and “I feel like I am taking all the right precautions to reduce my risk of getting coronavirus.” Participants who reported currently or previously having cancer were asked to rate an additional statement: “Having (or having had) cancer makes me feel particularly concerned about the coronavirus.” Agreement with each statement was again defined as selecting a 6 or 7, while disagreement was defined as selecting a 1 or 2.

##### Other COVID-19–Related Information Sources and Needs

To identify other sources this population has turned to for information during the pandemic, participants were asked, “What sources are you using to learn more about the novel coronavirus (COVID-19)?” and prompted to select all that apply from a list of 15 items (eg, internet search engines, social networking sites, or TV news reports). Finally, participants were asked to indicate the information or support that would be most helpful to them right now by selecting up to three choices from a list of 10 items such as “Information/guidance from my doctor about COVID-19 and my health condition/its treatment,” “emotional and/or mental health support,” and “Financial support for other necessities/bills (e.g., food, rent, etc.).” The full list of item responses can be found in Multimedia Appendix 1.

#### Demographic and Disease Characteristics

Participants were asked to indicate their age from a dropdown menu ranging from “Under 18” to “90 or older,” with each age in between listed continuously (eg, 18, 19, 20, ...). Participants were also asked to indicate their residence type (ie, rural, urban, or suburban), gender, highest level of education attainment, annual household income, and primary health insurance from categorical lists of item responses (see Multimedia Appendix 1). Participants indicated their chronic condition by selecting all that apply from a list of 25 chronic conditions, or participants were able to respond “none of these.” The full list of chronic conditions can be found in Multimedia Appendix 1, but examples include asthma, cystic fibrosis, Chron disease, migraines, and hypertension. Participants were also asked to indicate if they had been diagnosed with any from a list of nine types of cancer or “other” if they had been diagnosed with a cancer that was not listed.

### Analyses

Descriptive analyses were conducted to assess telehealth use, resource needs, and information sources among all participants, and *z* tests were used to explore differences between demographic groups. To assess for meaningful differences between demographic groups, age and education were each categorized into three groups (<40 years, 40-55 years, and ≥56 years, and high school, GED [General Educational Development], or less than high school; college degree; trade or vocational training; and some college, graduate, or professional degree), and income was categorized into four groups (<US $30,000, US $30,000-US $54,999, US $55,000- US $99,999, and ≥US $100,000). *Z* tests were also used to explore telehealth use by diagnosis and patient concerns.

## Results

### Participants

Among those who received information about the survey via email (n=98,983), 1758 (1.8%) people completed it, and an additional 452 people participated after finding information about the survey on one of the health community websites. Of the 2210 total survey respondents, 81% (n=1781) were female, 68% (n=1500) were older than 55 years, and 87% (n=1920) completed at least some college or trade school. The most commonly reported chronic conditions were hypertension (n=863, 39%), hyperlipidemia (n=562, 25%), asthma (n=424, 19%), and migraine (n=420, 19%), and 98% (n=2164) of respondents reported having health insurance. Approximately 30% (n=660) of respondents reported either full- or part-time employment, and 3% (n=75) were currently seeking employment. The remaining respondents were retired (n=849, 38%), stay-at-home parents (n=71, 4%), on disability (n=456, 21%), unemployed and not looking for employment (n=81, 4%), or currently in school (n=18, 1%). Among respondents who reported household incomes (n=1877), the majority (n=1507, 80%) reported annual household incomes under US $100,000. Table 1 displays full demographic and disease characteristics of the study sample.

**Table 1:** Sample demographic and chronic condition diagnoses.

Demographic			Participants (N=2210), n (%)
**Age (years)**			
	<40	224 (10.14)	
	40-55	486 (21.99)	
	≥56	1500 (67.87)	
**Gender**			
	Female	1781 (80.59)	
	Male	424 (19.19)	
	Nonbinary/gender nonconforming	5 (0.23)	
**Household income (US $; n=1877)**			
	<30,000	530 (28.24)	
	30,000-54,999	428 (22.80)	
	55,000-99,999	549 (29.25)	
	≥100,000	370 (19.71)	
**Education**			
	High School/GED^a^ or less than high school	290 (13.12)	
	College degree, trade/vocational training, or some college	1454 (65.79)	
	Graduate or professional degree	466 (21.09)	
**Employment status**			
	Retired	849 (38.42)	
	Employed	660 (29.86)	
	On disability	456 (20.63)	
	Not employed outside of the home	245 (11.09)	
**Health insurance**			
	Medicare	1040 (47.06)	
	Private, Employer-provided, or Health Insurance Exchange	964 (43.62)	
	Medicaid	130 (5.88)	
	Other/not sure	30 (1.36)	
	Do not have	46 (2.08)	
**Chronic condition**			
	High blood pressure	863 (39.05)	
	High cholesterol/hyperlipidemia	562 (25.43)	
	Asthma	424 (19.19)	
	Migraine	420 (19.00)	
	Multiple sclerosis	300 (13.57)	
	Rheumatoid arthritis	315 (14. 25)	
	COPD^b^/emphysema/chronic bronchitis	394 (17.83)	
	Irritable bowel syndrome	333 (15.07)	
	Type 2 diabetes	252 (11.40)	
	Psoriatic arthritis	169 (7.65)	
	Atopic dermatitis/eczema	111 (5.02)	
	Endometriosis	155 (7.01)	
	Crohn disease	147 (6.65)	
	Parkinson disease	130 (5.88)	
	Plaque psoriasis	122 (5.52)	
	Lupus	82 (3.71)	
	Macular degeneration	125 (5.66)	
	Ankylosing spondylitis	91 (4.12)	
	Heart failure	122 (5.52)	
	Ulcerative colitis	98 (4.43)	
	Hepatitis C	51 (2.31)	
	Axial spondyloarthritis/nonradiographic axial spondylarthritis	22 (1.0)	
	Cystic fibrosis	47 (2.13)	
	HIV	20 (0.90)	
	Alzheimer disease	4 (0.18)	
	Cancer	791 (35.79)	
	None	115 (5.20)	

^a^GED: General Educational Development.

^b^COPD: chronic obstructive pulmonary disease.

### Telehealth Engagement and Satisfaction

Among the 2210 survey respondents, 1073 (49%) respondents reported participating in a virtual visit (telehealth) with a doctor or health care provider (HCP) in the past 4 months, while 997 (45%) canceled or postponed regularly scheduled visits with their HCP, and 809 (37%) cancelled or postponed routine medical tests or planned to do so in the near future. Of those who used telehealth services (n=1073), 68% (n=725) agreed with the statement “I had a positive experience using telehealth.” Furthermore, 39% (n=420) agreed that “the virtual visit was just as good (or better) than an in-person visit,” but 14% (n=145) agreed with the statement “the technology was difficult to use.”

**Figure 1:**
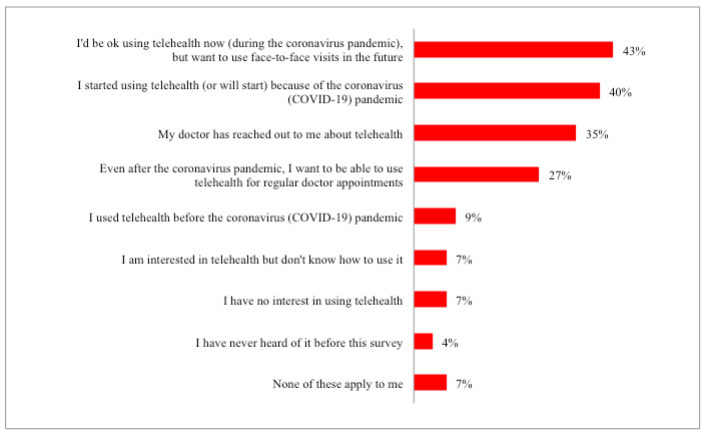
Participant’s self-reported experience using telehealth (N=2210).

As displayed in Figure 1, 40% (n=886) of the 2210 participants started using or planned to use telehealth options because of the COVID-19 pandemic, and 9% (n=194) reported engaging in telehealth options before the pandemic. Almost half of the participants (n=945, 43%) wanted to return to face-to-face appointments in the future, but 27% (n=591) reported a desire to use telehealth even after the pandemic subsides. Finally, 7% (n=162) of participants reported an interest in telehealth but did not know how to use it, while 4% (n=82) had never heard the term “telehealth,” and 7% (n=162) reported no interest in using telehealth.

**Figure 2:**
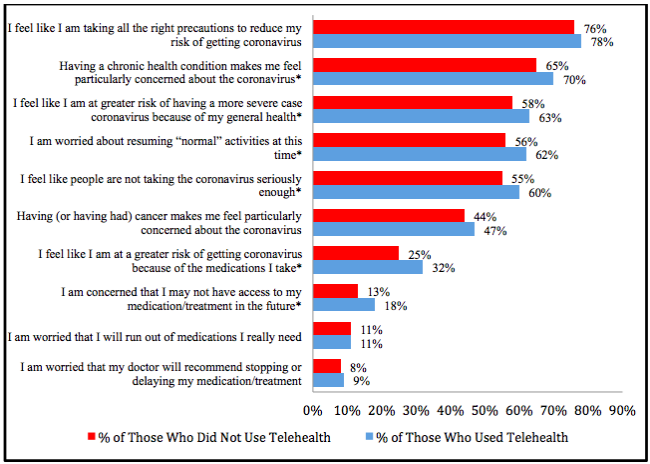
Participant concerns among those who used telehealth and did not use telehealth. *P<.05.

Looking at telehealth use by disease characteristics and patient concerns, patients with cystic fibrosis (n=33/47, 70%), lupus (n=55/82, 67%), and ankylosing spondylitis (n=60/91, 66%) recorded the highest proportions of patients using telehealth in the past 4 months when compared to patients with other diagnoses. When compared to those who did not engage in telehealth, higher proportions of those who used telehealth services reported feeling at higher risk of severe disease due to COVID-19 as indicated by agreeing with the statements “I feel like I am at a greater risk of getting coronavirus because of the medications I take” (341/1073, 32% vs 288/1137, 25%; *P*=.001) and “I feel like I am at a greater risk of having a more severe case of coronavirus because of my general health” (676/1073, 63% vs 659/1137, 58%; *P*=.02). Higher proportions of those who engaged in telehealth also agreed with the statements “Having a chronic health condition makes me feel particularly concerned about the coronavirus” (721/1024, 70% vs 698/1071, 65%; *P*=.01), “I am concerned that I may not have access to my medication/treatment in the future” (194/1073, 18% vs 153/1137, 13%; *P*=.003), and “I feel like people are not taking the coronavirus seriously enough” (646/1073, 60% vs 630/1137, 55%; *P*=.02). Figure 2 displays percentages of those who indicated various concerns by telehealth use status.

#### COVID-19 Information Sources and Needs

##### COVID-19 Communications With a Health Care Team

Of the 2210 participants, 72% (n=1592) reported communicating with their health care team about COVID-19 in some manner. A complete list of the ways in which this discussion was initiated is listed in Multimedia Appendix 2; however, the most highly endorsed responses were “I reached out via phone, online portal, etc.” (n=800, 36%) and “My doctor’s office sent out information and recommendations (via email, online portal, letter, social media” (n=602, 27%). The most common modes of communication among those who had spoken with an HCP about the virus were by phone (n=1183, 74%), in a telehealth appointment (n=705, 44%), and via online portal (n=680, 43%). Primary topics of these communications were decisions regarding treatment regimens (continuing as prescribed: n=933, 60%; changing: n=181, 12%; or temporarily stopping: n=119, 8%), regular check-ups (n=853, 55%), CDC COVID-19 guidelines (n=660, 42%), and decisions to cancel or delay upcoming appointments and surgeries (n=445, 29%). A complete list of topics discussed can be found in Multimedia Appendix 2.

##### Other COVID-19–Related Information Sources and Needs

HCPs were not the only source of information respondents turned to in response to hearing about COVID-19. When asked what sources respondents used to learn more about the virus, 63% (n=1389) selected TV news reports, 58% (n=1284) chose news websites, 57% (n=1268) turned to government websites, and 40% (n=892) reported retrieving information through search engines such as Yahoo or Google. In total, 60% (n=1333) of respondents reported that they either already looked up information online or planned to in response to hearing about COVID-19.

When asked what information or support would be most helpful right now, just over half of the survey respondents (n=1151, 52%) answered “understanding how COVID-19 affects people with my health condition,” 48% (n=1071) indicated obtaining up-to-date and accurate information about the virus, 24% (n=538) selected emotional or mental health support, and 22% (n=485) indicated that receiving information from their HCP about COVID-19 and their health condition would be helpful. Additionally, 19% (n=412) indicated a need for financial support for life necessities such as food and rent. Significantly higher proportions of those who engaged in telehealth indicated a need for the following types of support than those who did not engage in telehealth: information from the company who makes my medications about COVID-19 and the treatment I take (126/1073, 12% vs 94/1137, 8%; *P*=.006), emotional or mental health support (292/1073, 27% vs 246/1137, 22%; *P*=.002), and financial support for medications and health care costs (167/1073, 16% vs 132/1137, 12%; *P*=.007). Finally, 16% (n=359) of the 2210 respondents indicated no need for additional information or support.

#### Demographic Comparisons

In this section, we highlight notable trends and differences in telehealth engagement, satisfaction, information sources, and needs between members of various demographic backgrounds. A full list of comparisons can be found in Multimedia Appendix 2.

##### Telehealth Engagement and Satisfaction

When looking at telehealth engagement across sociodemographic categories, a higher proportion of women engaged in telehealth than men (890/1781, 50% vs 181/424, 43%; *P*=.007), and a higher proportion of those earning household incomes over US $100,000 engaged in telehealth more than those earning less than US $30,000 (195/370, 53% vs 241/530, 45%; *P*=.03). The proportion of those engaging in telehealth also increased across educational attainment levels, such that a greater percentage of those who completed at least some college or trade school engaged in telehealth than those who completed high school or below (706/1454, 49% vs 114/290 39%; *P*=.004), and a greater percentage of those who completed an advanced degree engaged in telehealth than those who completed college or trade school (253/466, 54% vs 706/1454, 49%; *P*=.03). Although 59% (133/224) of those younger than 40 years and 54% (263/486) of those aged 40-55 years used telehealth, a smaller proportion of aging populations did so, with only 45% (677/1500) of individuals 56 years or older reporting telehealth use (*P*<.001 and *P*=.001, respectively). Telehealth use did not significantly differ across residence types (rural, suburban, or urban).

Higher proportions of female telehealth users found the virtual visit to be just as good or better than an in-person visit compared to males (360/890, 40% vs 58/181, 32%; *P*=.04), and a higher proportion of those with a high school degree or below also found the virtual visit to be just as good or better than an in-person visit compared to those with advanced degrees (54/114, 47% vs 91/253, 36%; *P*=.04). When comparing telehealth satisfaction among various income groups, higher percentages of those with lower household incomes (<US $30,000: 100/241, 41%; US $30,000-US $54,999: 86/208, 41%; and US $55,000-US $99,999: 108/266, 41%) also found the virtual visit to be just as good or better than an in-person visit when compared to those earning over US $100,000 annually (59/195, 30%; all *P*=.02). A greater percentage of individuals with advanced degrees disagreed with the statement “the technology was difficult to use” (184/253, 73%) compared to those who reported lower educational levels (at least some college or trade school: 441/706, 62%; *P*=.003; high school or below: 69/114, 61%; *P*=.02). Finally, a greater percentage of those in suburban areas also disagreed with this statement compared to those living in urban areas (372/549, 68% vs 135/224, 60%; *P*=.047).

A significantly larger proportion of those who were 56 years or older reported no interest in using telehealth (128/1500, 9%) compared to younger age groups (25/486, 5% of those aged 40-55 years; *P*=.02; 9/224, 4% of those younger than 40 years; *P*=.02) and desired to return to in-person appointments after the pandemic (675/1500, 45% vs 192/486, 40% of those aged 40-55 years; *P*=.03; and 78/224, 35% of those younger than 40 years; *P*=004). Higher proportions of those 56 years or older also rated the technology difficult to use (107/677, 16%, compared to 12/133, 9% of those younger than 40 years; *P*=.04; and 26/263, 10% of those aged 40-55 years; *P*=.02), but there were no significant differences between age groups in their agreement with the statement “I feel like the virtual visit was just as good (or better) than an in-person visit.” Furthermore, a larger percentage of the older age group were also interested in telehealth but did not know how to use it (125/1500, 8% vs 27/486, 6% of those aged 40-55 years; *P*=.045, and 10/224, 4% of those younger than 40 years; *P*=.04).

##### COVID-19 Information Sources and Needs

###### COVID-19 Communications With a Health Care Team

When looking at engagement with a health care team regarding COVID-19 information sources and needs, it may be notable that smaller proportions of those who earned a high school degree or less reported that their doctor’s office sent out information and recommendations about the virus when compared to peers who reported higher educational attainment (53/290, 18% vs 397/1454, 27% of those who completed at least some college or trade school; *P*=.001; and 152/466, 33% who held an advanced degree; *P*<.001). Similarly, smaller proportions of individuals with household incomes under US $30,000 (134/530, 25%) and between US $30,000 and US $54,999 (109/428, 25%) reported such a distribution of information compared to those earning at least US $100,000 (122/370, 33%; *P*=.01 and *P*=.02, respectively). Furthermore, higher proportions of those younger than 40 years (113/224, 50%) and between 40 and 55 years (211/486, 43%) reached out to their HCP than those older than 55 years (476/1500, 32%; both *P*<.001). Even when meeting with an HCP, a smaller percentage of those older than 56 years discussed monitoring for COVID-19 symptoms (140/1039, 13% vs 63/350, 18% of those 40-55 years; *P*=.04; and 36/171, 21% of those younger than 40 years; *P*=.009) and what to do if experiencing COVID-19 symptoms (199/1039, 19% vs 106/350, 30% of those aged 40-55 years; *P*<.001; and 52/171, 30% of those younger than 40 years; *P*=.001) compared to younger age groups (see Multimedia Appendix 2).

###### Other COVID-19–Related Information Sources and Needs

Looking at which COVID-19 information sources were most popular among various demographic groups, government websites (eg, CDC, National Institutes of Health, and local health departments) were the most popular selection among participants younger than 40 years (n=165, 74%), aged 40-55 years (n=329, 68%), and those with annual household incomes over US $100,000 (n=240, 65%). TV news reports were most popular among those 56 years or older (n=1009, 67%), all those earning under $100,000 annually, and all those reporting education levels up to a college degree. They were also the most popular choice regardless of residence type and gender (see Multimedia Appendix 2). Finally, news websites were the most popular choice among those with an advance degree (n=322, 69%).

“Understanding how COVID-19 affects people with my health condition” was of primary interest across all age groups, residence types, education levels, and household incomes, with the exception of those with a high school degree or below, among whom a slightly higher percentage (135/290, 47% vs 134/290, 46%) indicated a general need for up-to-date and accurate information about COVID-19. There were significant differences in information and support needs between age groups such that higher percentages of those younger than 40 years expressed interest in the following resources compared to older age groups: financial support for necessities (69/224, 31% vs 215/1500, 14% of those 56 years or older; *P*<.001); learning how to have food, medication, or supplies delivered (29/224, 13% vs 34/486, 7% of those aged 40-55 years; *P*=.01; and 112/1500, 7% of those 56 years or older; *P*=.005); and emotional or health support (84/224, 38% vs 138/486, 28% of those aged 40-55 years; *P*=.02; and 316/1500, 21% of those 56 years or older; *P*<.001). Conversely, a higher percentage of those 56 years or older stated that they were not in need of any more information or support (280/1500, 19%) compared to those aged 40-55 years (63/486, 13%; *P*=.004) and younger than 40 years (16/224, 7%; *P*<.001).

When examining information and resource needs across other demographic factors, a significantly larger proportion of females also expressed a need for emotional or mental health support than males (465/1781, 26% vs 71/424, 17%; *P*<.001). A smaller proportion of those with an advanced degree expressed a need for financial support compared to those of lower educational attainment, both for medications and health care expenses (44/466, 9% vs 214/1454, 15% of those with some college or trade school; *P*=.004; and 41/290, 14% of those with a high school degree or below; *P*=.047), and for other necessities and bills (59/466, 13% vs 283/1454, 19% of those with some college or trade school; *P*=.001; and 70/290, 24% of those with a high school degree or below; *P*<.001). Finally, the need for information regarding COVID-19 in general and its relation to one’s health condition and current treatments tended to increase as household income increased, while financial and food medication delivery information needs tended to decrease (see Multimedia Appendix 2).

## Discussion

### Principal Results

The purpose of this study is to assess the telehealth use, resource needs, and information sources of individuals living with chronic conditions during the COVID-19 pandemic. According to these results, 45% (n=997/2210) of the study sample either cancelled or planned to cancel appointments with their HCP during times of COVID-19, and nearly half (1073/2210, 49%) engaged in telehealth appointments in the 4 months prior to data collection. Although 40% (886/2210) of participants initiated telehealth care because of the pandemic, 7% (162/2210) responded that they were interested in telehealth and did not know how to use it, 7% (162/2210) reported that they had no interest in using telehealth, 4% (82/2210) reported they had never heard the term prior to taking the survey, and only 35% (780/2210) of patients reported that their doctor reached out to them about telehealth. Thus, as recommendations continue for patients with underlying medical conditions to mitigate possible exposure outside of the home and as practices look to extend telehealth services beyond the pandemic [11], providers need to ensure adequate information is distributed to inform patients about telehealth procedures. A recent survey of 2949 adults living in China found that high-quality communication with a provider prior to COVID-19 was associated with preventive behavior uptake during the pandemic [14]. Distributing patient-centered, personalized communications about telehealth from HCPs may help increase uptake of telehealth and maintain continuity of care in the future.

It may also be important to note that just under half of survey respondents (945/2210, 43%) were content using telehealth services during the pandemic but desired to return to in-person care in the future. With this in mind, information provided to patients about telehealth may also include information about future plans to return to in-person visits, including public and personal health standards that need to be met to do so. In the interim, providers may also consider implementing recently suggested strategies to enhance feelings of “copresence” or connection between patient and provider during telehealth visits [7,15]. Such strategies include postvisit mood evaluations, one-click responses from health care teams, and encouraging messages, and may help further engage patients, alleviate stress, and maintain continuity of care during the pandemic [7,15].

As telehealth outreach increases, HCPs can optimize virtual visits by addressing patients’ top concerns during the pandemic. When asked what support or information would be most helpful, the top-rated responses included information about COVID-19, how it impacts individuals with specific diagnoses and disease regimens, and mental or emotional health support. Experts have already noted the important role telehealth could play in providing remote interdisciplinary care [16], including mental and emotional health services [17] during this global crisis. Thus, telehealth services across multiple disciplines and hospital services, including mental health care, social work, and patient navigation, may prove vital to providing quality care and meeting all information and supportive care needs of patients during COVID-19.

Finally, it is imperative to note which groups may be at highest risk of losing access to care as services shift to digital formats during COVID-19. According to these results, greater proportions of adults younger than 56 years have engaged in and are interested in telehealth than those 56 years or older, which may be unsurprising given the reported digital divide between younger and older generations [18]. This phenomenon has also been reflected in other literature assessing telehealth use both prior to and during the pandemic [19-21]. Although a higher percentage of older adults expressed no interest in telehealth compared to younger age groups, nearly half of those 56 years or older started using telehealth in response to COVID-19, nearly half said they would be willing to participate in telehealth even though they wanted to return to in person in the future, one-third wanted to participate in telehealth even after the pandemic, and only 5% reported being interested but not knowing how to use telehealth. Given the fact that older adults, with or without underlying medical conditions, are at higher risk of serious illness from COVID-19 [1,22-24], particular efforts to communicate the benefits and importance of telehealth during the pandemic and engage this group in telehealth efforts may be vital. Because lower percentages of aging adults reported reaching out to their health care provider about COVID-19, it may also be particularly important for health care providers to initiate conversations about telehealth and pandemic-related information.

Similarly, smaller percentages of participants who reported lower household incomes and educational attainment participated in telehealth when compared to peers of higher income and educational status, realities that have also been noted in previous literature on digital health use [18,20,25]. Other researchers have noted measures HCPs can take to try to ensure telehealth does not exacerbate health inequities during the pandemic (eg, ensuring language interpreter access, recruiting telehealth staff from diverse backgrounds, providing patient trainings in telehealth use, and informing patients about free or discounted broadband access) [25,26]. Thus, future outreach efforts should focus on equitable communication and engagement of patients in telehealth practices.

### Limitations

This study presents important information regarding telehealth use, resource needs, and information sources of individuals considered at risk for severe disease during COVID-19 due to underlying medical conditions. This data collection is not without limitations. Given the exploratory nature of this study, many of these measures have not been validated, and the general description of telehealth as a “virtual visit with a doctor/healthcare professional” may not encompass the full scope of telehealth practices taking place during the COVID-19 pandemic. Despite this fact, only 4% of individuals in the study reported that they had never heard of telehealth prior to the study, indicating a general awareness if the terminology among the study population. Because all participants were members of online health communities, the telehealth and information-seeking practices of this sample may also be skewed, as participants may be more comfortable with digital health solutions than the general public.

Finally, at approximately 2%, the response rate for this survey was low, the sample was primarily female (1781/2210, 81%), and the data was skewed toward older populations who rated their conditions with higher severity, which may skew the age analyses presented and limit the generalizability of these results. With this said, the demographic characteristics of this sample do mirror those of the larger sampling frame, given that, among 36,515 survey responses collected from American Health Union members over the previous year, 81% of responses were female and 48% were 60 years or older. By leveraging existing online health communities for data collection, however, this study included a large, national sample of individuals with underlying medical conditions and provides preliminary guidance regarding the telehealth practices and needs of this high-risk population during the COVID-19 pandemic.

### Conclusions

These results offer important insight to current telehealth practices and information needs of patients with underlying medical conditions during COVID-19. Although many patients are engaging in telehealth, they continue to seek information about COVID-19, how the virus impacts people with their particular condition and treatment regimen, and support for mental and emotional health during the pandemic. Moving forward, medical and public health professionals may continue to take an active approach to engaging patients with underlying conditions in available telehealth services, particularly those who are members of lower socioeconomic status and aging populations. Future research will explore changes in telehealth practices and information needs over time by examining data from multiple waves of this survey distributed between March and July of 2020.

## Appendix

Multimedia Appendix 1 Measures.

Multimedia Appendix 2 Demographic comparisons.

## Abbreviations

CDC: Centers for Disease Control and Prevention
GED: General Educational Development
HCP: health care provider
