# Supplementary material for: Telemedicine Use and Health-Related Concerns of Patients With Chronic Conditions During COVID-19: Survey of Members of Online Health Communities
Source: J Med Internet Res. 2021 Feb 18;23(2):e23795. doi: 10.2196/23795 (PMC7894395; doi:10.2196/23795)
Supplement: Multimedia Appendix 1 [file jmir_v23i2e23795_app1.docx]

# Appendix 1: Measures

## Telehealth Engagement and Satisfaction

Have you had a “virtual visit” (telehealth) with your doctor / healthcare professional in the past 4 months?

- Yes
- No

IF USED TELEHEALTH

Please rate your agreement with the statements below about your experience with “virtual visits” (telehealth) with your doctor / healthcare professional.

| RANDOMIZE | **Completely disagree** | | |  | | | **Completely**  **agree** | | |
| --- | --- | --- | --- | --- | --- | --- | --- | --- | --- |
| I had a positive experience using telehealth | 1 | 2 | 3 | | 4 | 5 | | 6 | 7 |
| The technology was difficult to use | 1 | 2 | 3 | | 4 | 5 | | 6 | 7 |
| I feel like the virtual visit was just as good (or better) than an in-person visit | 1 | 2 | 3 | | 4 | 5 | | 6 | 7 |

You may have heard about virtual appointments with doctors (sometimes called “telehealth”) that may take place over video chat. Which statements below about telehealth apply to you? (Select all that apply)

- - I have never heard of it before this survey
  - My doctor has reached out to me about telehealth
  - I am interested in telehealth but don’t know how to use it
  - I have no interest in using telehealth
  - I used telehealth before the coronavirus (COVID-19) pandemic
  - I started using telehealth (or will start) because of the coronavirus (COVID-19) pandemic
  - Even after the coronavirus pandemic, I want to be able to use telehealth for regular doctor appointments
  - I’d be ok using telehealth now (during the coronavirus pandemic), but want to use to face-to-face visits in the future
  - None of these apply to me

### COVID-19 Information Sources and Needs

In which of the following ways have you communicated with your doctor / healthcare team about the novel coronavirus (COVID-19)? (Select all that apply)

- - - I reached out via phone, online portal, etc
    - My doctor personally contacted me (via phone, online portal, email)
    - A member of my healthcare team (nurse, etc) contacted me (via phone, online portal, email)
    - My doctor’s office sent out information and recommendations (via email, online portal, letter, social media)
    - We discussed it during a regular appointment
    - I made a *specific* appointment to discuss it
    - Other (specify _____)
    - I haven’t communicated with my doctor or healthcare team about coronavirus

IF HAVE COMMUNICATED WITH DOCTOR/HCP

How have you communicated with your doctor / healthcare professional? (Select all that apply)

- - By phone
  - By portal
  - By email
  - In a telehealth appointment (video chat)
  - At an in-person appointment at their office
  - In-person in a hospital, infusion center, etc
  - Other (specify _____)
  - I have not heard back / they reached out to me and I have no need to contact them

IF HAVE COMMUNICATED WITH DOCTOR/HCP

What did you discuss with your doctor / healthcare professional? (Select all that apply)

- Deciding to **stay on my current treatment plan** as prescribed / not stop or skip medication
- Deciding to **temporarily stop or skip** my current medication or treatment
- Deciding to **change my medication / treatment** to something else
- Following of CDC guideline/precautions (eg, stay home, frequent hand washing, wearing a mask and gloves)
- Monitoring for novel coronavirus (COVID-19) symptoms
- What to do if experiencing novel coronavirus (COVID-19) symptoms (or if symptoms became worse)
- Advice/guidance/precautions for resuming some normal activities, such as returning to work
- Deciding to **cancel / delay an upcoming appointment** and/or surgery
- **Regular discussion / check-in** about my current condition / symptoms
- **Experiencing illness/symptoms** for something **other than my current condition or COVID-19** (eg, ear infection, UTI)
- Other (specify _____)

What, if anything, **have you done** – or are you **planning to do** in the near future -- in response to hearing about the novel coronavirus (COVID-19)? (Select all that apply)

- - - Contact my doctor / healthcare team (via phone, online portal, etc)
    - Look up information online
    - Avoid large gatherings
    - “Social distancing” (eg, avoiding shaking hands, hugs, close personal contact, etc)
    - Stay home more often / avoiding public places (eg, movies, restaurants, etc)
    - Start to work remotely / from home
    - Stock up on medication
    - Stop taking or skip medication
    - Start getting medication delivered to my home
    - Contact a patient services / support program for my medication
    - Wash hands more frequently / for longer
    - Change (or cancel) travel plans
    - Cancel or postpone regular doctor/healthcare visits
    - Make a specific visit / appointment to see my doctor/healthcare professional
    - Have a “virtual” visit with a doctor/healthcare professional – “telehealth” – via video chat
    - Go to the ER
    - Stop working or reduce my hours (by my choice or not by my choice)
    - Wearing personal protective equipment (PPE) outside my home (eg, gloves, mask)
    - Contact a doctor because of possible coronavirus exposure or symptoms
    - Clean, disinfect, and/or sanitize my home and/or personal items
    - Cancel / postpone routine medical tests, like blood draws, MRIs, CT scans
    - Other (specify _____)
    - I have not done anything in response to coronavirus

Please rate your agreement – **from your point of view –** with the following statements below about the novel coronavirus (COVID-19):

| RANDOMIZE | **Completely disagree** | | |  | | | **Completely**  **agree** | | |
| --- | --- | --- | --- | --- | --- | --- | --- | --- | --- |
| [IF HAVE ANY QUALIFY CONDITION LISTED IN S5]  Having a **chronic health condition** makes me feel particularly concerned about the coronavirus | 1 | 2 | 3 | | 4 | 5 | | 6 | 7 |
| [IF HAVE ANY CANCER IN S6]  Having (or having had) **cancer** makes me feel particularly concerned about the coronavirus | 1 | 2 | 3 | | 4 | 5 | | 6 | 7 |
| I feel like people are not taking the coronavirus seriously enough | 1 | 2 | 3 | | 4 | 5 | | 6 | 7 |
| I am worried that I will run out of medications I really need | 1 | 2 | 3 | | 4 | 5 | | 6 | 7 |
| I feel like I am at a **greater risk of** **getting** coronavirus because of the **medications I take** | 1 | 2 | 3 | | 4 | 5 | | 6 | 7 |
| I feel like I am at greater risk of **having a more severe case** coronavirus because of my **general health** | 1 | 2 | 3 | | 4 | 5 | | 6 | 7 |
| I feel like I am taking all the right precautions to reduce my risk of getting coronavirus | 1 | 2 | 3 | | 4 | 5 | | 6 | 7 |
| I am worried that my doctor will recommend stopping or delaying my medication/treatment | 1 | 2 | 3 | | 4 | 5 | | 6 | 7 |
| I am concerned that I may not have access to my medication/treatment in the future | 1 | 2 | 3 | | 4 | 5 | | 6 | 7 |
| I am worried about resuming “normal” activities at this time | 1 | 2 | 3 | | 4 | 5 | | 6 | 7 |

What sources **are you using** to learn more about the novel coronavirus (COVID-19)? (Select all that apply)

- - - Internet search engines like Yahoo or Google
    - Websites designed for healthcare professionals (eg, academic journals)
    - Government websites (eg, CDC, NIH, local health department)
    - General health websites (for example, WebMD, iVillage, etc)
    - Condition-specific websites (eg, National MS Society, Arthritis Foundation, American Cancer Society)
    - Pharmaceutical company websites for medications I’m taking
    - Social Networking sites such as Facebook and Twitter
    - Video-oriented websites like YouTube
    - Online blogs, forums or support communities
    - News websites (eg, CNN.com, MSNBC.com, FoxNews.com)
    - TV news reports
    - Special TV programs about coronavirus
    - Newspapers
    - Other (specify _____)
    - I have not used any sources to learn more

What types of information and/or support would be most helpful to you right now? (Select up to 3 that would be most helpful to you)

- Information/guidance **from my doctor** about COVID-19 and my health condition / its treatment
- Information/guidance **from the company who makes my medication(s)** about COVID-19 and the treatment I take
- **Up-to-date and accurate information** about COVID-19, like its spread, its treatment, signs/symptoms, preventive measures, etc
- **Emotional** and/or mental health **support**
- **Financial** support to pay for my **medications / healthcare costs**
- **Financial** support for **other necessities** / bills (eg, food, rent, etc)
- Understanding how COVID-19 **affects people with my health condition(s)**
- Information about making sure my **medication will be available to me**
- Learning how to have food, medication and/or supplies **delivered to my home**
- I am not in need of any more information and/or support

## Demographics and Disease Characteristics

1. Have you been formally diagnosed by a healthcare professional with any of the following conditions? (Select all that apply)

- Alzheimer’s disease
- Ankylosing spondylitis (AS)
- Asthma
- Atopic dermatitis / eczema
- Axial spondyloarthritis / non-radiographic axial spondyloarthritis
- COPD / emphysema / chronic bronchitis
- Crohn’s disease
- Cystic fibrosis
- Endometriosis
- Heart failure
- Hepatitis C
- High blood pressure / hypertension
- High cholesterol / hyperlipidemia
- HIV
- Irritable bowel syndrome (IBS)
- Lupus
- Macular degeneration
- Migraine
- Multiple sclerosis (MS)
- Parkinson’s disease
- Plaque psoriasis
- Psoriatic arthritis (PsA)
- Rheumatoid arthritis (RA)
- Type 2 diabetes
- Ulcerative colitis (UC)
- None of these

1. Have you ever been diagnosed with any of the following cancers? (Select all that apply)

- Bladder cancer
- Blood cancer (eg, leukemia, lymphoma, multiple myeloma, MDS)
- Breast cancer
- Colon cancer (colorectal cancer)
- Head or neck cancer
- Kidney cancer (renal cancer)
- Lung cancer
- Prostate cancer
- Skin cancer (eg, melanoma, basal cell carcinoma, squamous cell carcinoma)
- Other type of cancer (please specify): _________
- I have not been diagnosed with any cancers

1. What is your age?

<Drop-down menu with options ranging from Under 18, 18, 19, 20 … to 89, 90 or older>

1. Which of the following best describes where you live?
   - Large city / urban area
   - Suburbs around a city / urban area
   - Rural area
2. What is your gender?
   - Female
   - Male
   - Non-binary/ Gender non-conforming
3. What is the highest level of education you have completed?

- No schooling
- No schooling past 8th grade
- Some high school, did not graduate
- High school graduate, diploma, or the equivalent (eg, GED)
- Trade/technical/vocational training
- Some college, no degree
- 2-year college degree (Associate's degree)
- 4-year college degree (Bachelor's degree)
- Master’s degree
- Doctoral or professional degree (PhD, MD, JD)

1. What is your annual household income?

- Less than $30,000
- $30,000 - $54,999
- $55,000 - $74,999
- $75,000 - $99,999
- $100,000 - $149,999
- $150,000 - $199,999
- $200,000 or more
- Prefer not to answer

1. What is your current employment status?

- Employed, full time
- Employed, part time
- Self-employed
- Unemployed, looking
- Unemployed, not looking
- On disability
- Fully retired
- Student
- Homemaker/Stay-at-home parent

1. What is your primary health insurance?

- Group coverage, through my employer or the employer of a spouse or family member
- Private insurance, purchased directly from the insurance company
- Health insurance exchange, enrolled through Affordable Care Act
- Medicare
- Medicaid
- Military coverage (DOD), VA, or TriCare
- Other insurance type/Not sure of what type
- Do not have
